# Characterization of the structural, physicochemical, and functional properties of soluble dietary fibers obtained from the peanut shell using different extraction methods

**DOI:** 10.3389/fnut.2022.1103673

**Published:** 2023-02-01

**Authors:** Lei Wang, Rui Fan, Yanhua Yan, Shuo Yang, Xuesong Wang, Baiqin Zheng

**Affiliations:** ^1^Tangshan Food and Drug Comprehensive Testing Center, Tangshan, China; ^2^Key Laboratory of Quality Evaluation and Nutrition Health of Agro-Products, Ministry of Agriculture and Rural Affairs, Tangshan, China; ^3^Hebei Agricultural Products Quality and Safety Testing Innovation Center, Tangshan, China; ^4^Tangshan Institute of Industrial Technology for Functional Agricultural Products, Tangshan, China; ^5^Department of Nutrition and Food Hygiene, School of Public Health, Peking University, Beijing, China

**Keywords:** peanut shell, soluble dietary fiber, physicochemical properties, functional properties, structure

## Abstract

**Objective:**

To propose a possible solution for a peanut by-product, peanut shell (PS), this study evaluated the effects of different methods, including enzymatic extraction (E-SDF), microwave extraction (M-SDF), and pulsed electric field extraction (PEF-SDF), on the characterization of soluble dietary fibers (SDFs) from PS.

**Methods:**

We determined the physicochemical properties, including water- and oil-holding capacities (WHC and OHC), emulsifying properties, rheological properties, functional properties, including pancreatic lipase activity inhibition (PRAI), glucose and cholesterol adsorption capacities (GAC and CAC), and the structural properties of SDFs.

**Results:**

The results showed that PEF-SDF possessed the highest WHC, OHC, and emulsifying properties. M-SDF and PEF-SDF appeared to have more complex and porous structures, and they showed small molecular weights. Notably, PEF-SDF showed the strongest capacities in CAC, GAC, and PRAI.

**Conclusions:**

The results indicate that PEF-SDF is a potential SDF preparation method for a promising dietary fiber (DF) source, PS.

## 1. Introduction

Peanuts have a consolidated tradition of cultivation and processing in the Asian and American continents and have a huge market (1). China relatively has the largest cultivation area and annual output of peanuts in the world and is relatively the largest producer and consumer of peanuts in the world (2). Peanuts are mainly used in peanut oil or refined foods, but the peanut industry produces large quantities of by-products, mainly peanut shell (PS), every year (3). PSs are rich in abundant nutrients, including protein (4.8%−7.2%), crude fat (1.2%−1.8%), multiple minerals (such as Ca, Fe, Cu, Zn, Mn, Na, P, K, and Mg), and bioactive substances, containing β-sitosterol and saponin. With fiber and hemicellulose contents being 65.7%−79.3% and 10.1%−11.6%, respectively, it is worth mentioning that PS is a good source of natural dietary fiber (DF) (4). The peanut processing industry, which is growing rapidly, which simulatenously increases the quantity of shells, was simply discarded or directly used as animal feed, which could cause a waste of available resources and possibly even environmental pollution (5). Therefore, it is important to realize how eco-friendly PS can be used to decrease waste and indirectly generate income. Indeed, PS has the potential to be an excellent choice for producing soluble dietary fibers (SDFs).

In recent decades, dietary fibers have been found to have many health benefits, including reducing the risks of heart disease, diabetes, obesity, and some forms of cancer, because they are resistant to digestion and absorption in the small intestine while allowing fermentation in the large intestine (6, 7). The World Health Organization (WHO) strongly recommends that adults consume at least 25 g of DF per day (8), while the majority of people worldwide consume <20 g/day (9). The survey reports that the intake of DF in the Chinese population is deficient, with <5% of the population being able to meet the appropriate intake (25 g/day). The use of PS as a source of DF, a functional or novel fiber, in various human foods could be promising for the future.

According to the solubility of DF in water, it can be divided into insoluble dietary fiber (IDF; hemicellulose, cellulose, and lignin) and SDF (pectin, β-glucans, glactomannans, fructans, oligosaccharides, some hemicelluloses, guar, gums, and mucilage) (10). Compared with IDF, which is completely hydrolyzed in the colon to promote probiotic growth, SDFs play an important role in lowering the glycemic reaction and plasma cholesterol and in reducing the risk of cardiovascular diseases (CVD) (11, 12). According to a previous report, the proportion of SDF in total dietary fiber (TDF) must be at least 10% to be recognized as high-quality DF (13). In addition, SDFs can be used as a fat substitute and flavor enhancer (14, 15). To further increase SDF yield and quality, emerging technologies based on the physical, biological, and chemical theories have been utilized, demonstrating the different advantages and limitations of various aspects of its properties (16, 17).

In this study, the effects of different extraction methods, including enzymatic extraction (E-SDF), microwave extraction (M-SDF), and pulsed electric field extraction (PEF-SDF), on the structural, physicochemical, and functional properties of SDFs were compared. The results provide technical support for high-value applications of PS from peanut production in the future.

## 2. Materials and methods

### 2.1. Materials and reagents

The PS purchased from Tangshan Runze Cereals, Oils, and Food Co. Ltd. was dried at −80°C for 24 h in a vacuum environment, crushed, and subsequently passed *via* an 80-mesh screen.

Glucose amylase (100 U/mg) and thermostable α-amylase (30 U/mg) were purchased from Solebo Biotech Ltd. (Beijing, China). Standard monosaccharides were purchased from Sigma-Aldrich (St. Louis, USA).

### 2.2. SDF extraction

**Figure 1:**
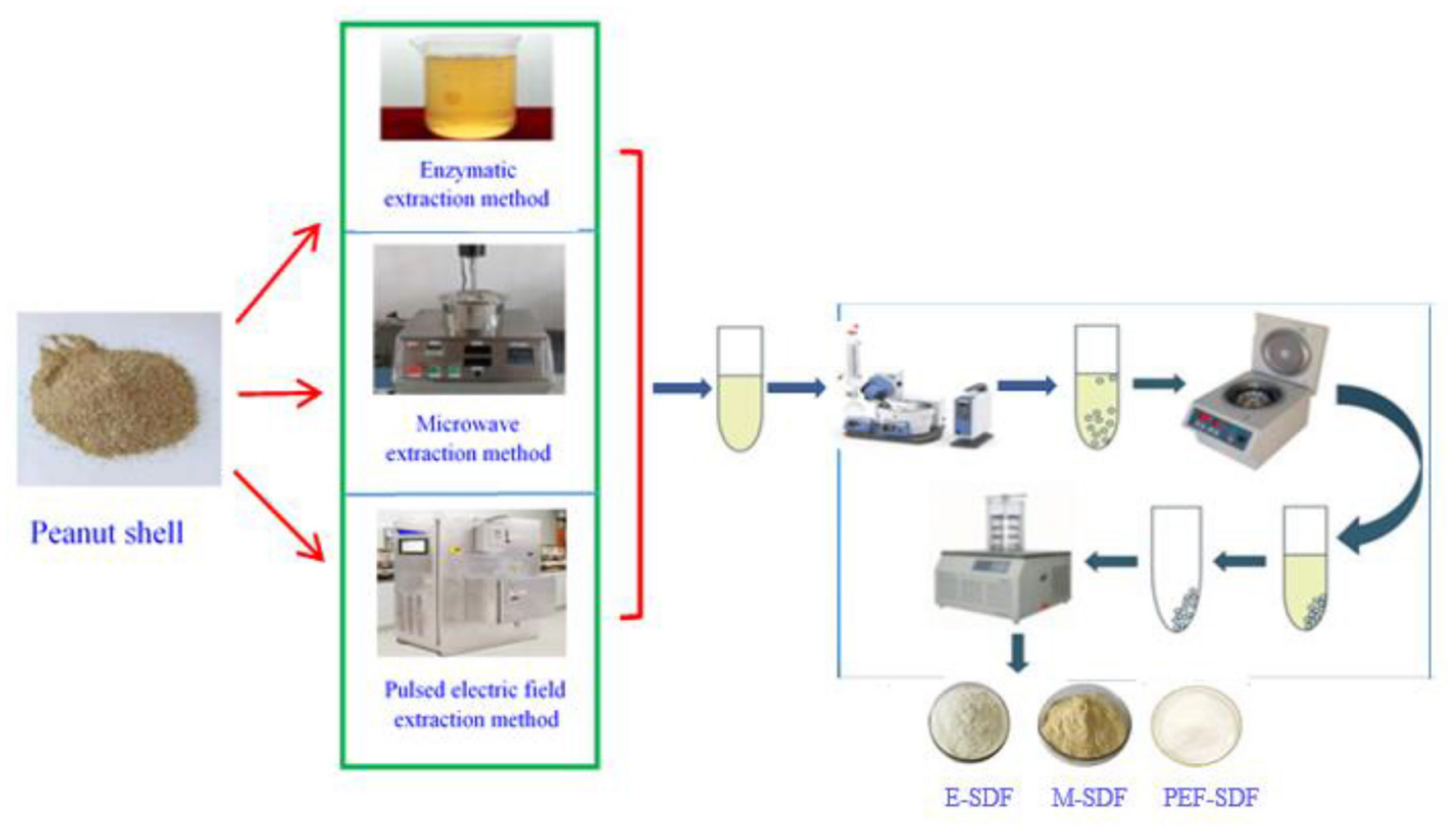
A scheme of soluble dietary fiber (SDF) extraction procedure.

Soluble dietary fiber preparation processes using three methods, namely enzymatic, microwave, and pulsed electric field-assisted extraction, are described in Figure 1.

#### 2.2.1. Method 1 (E-SDF)

Using Moczkowska's method (18), the dried PS sample (50 g) from 1,000 g of PS material was dissolved in the deionized water (1:20, w/v) and adjusted to pH 4.5 with polybutylene succinate (PBS). Approximately 0.8% of the mixed enzyme was added to the PS solution, stirred at 58°C for 1 h, inactivated for 9 min, centrifuged at 3,600 rpm/min for 8 min, and precipitated overnight with 4 times the volume of ethanol. The collected sediment was sufficiently dried to yield E-SDF.

#### 2.2.2. Method 2 (M-SDF)

Soluble dietary fibers were prepared based on the microwave method proposed by Kapusniak (19). The dried PS sample (50 g) from 1,000 g of PS material was dissolved in deionized water (1:25, w/v), and the pH value was adjusted to 4.5 with PBS. Protease was added to the sample and stirred at 58°C for 30 min, then placed in an ANKS-C1 microwave processor (ANKS Company, Qingdao, China) at 700 W for 5 min, and centrifuged and precipitated to obtain M-SDF. Detailed operations have been described earlier to obtain M-SDF.

#### 2.2.3. Method 3 (PEF-SDF)

According to Kim et al.'s method (20), 50 g of dried PS was weighed and mixed with 20 times the volume of distilled water at room temperature. The pH was adjusted to 4.5. Protease (0.6%, m/v) was added to the mixture, stirred at 60°C for 40 min, inactivated on a water bath at 100°C for 10 min, and then the parameters of electrical field intensity of 8 kV/cm, frequency of 1 Hz, and pulse width of 20 μs were set on an EX-1900 pulsed electric field device. Detailed operations have been described earlier to obtain PEF-SDF.

### 2.3. Physicochemical properties

#### 2.3.1. Water-holding capacity

Water-holding capacity (WHC) was tested in accordance with a previous report (21). The SDF (0.5 g) was dissolved in distilled water, kept at room temperature for 24 h, and centrifuged at 4,000 rpm for 20 min. The sediment was weighed, and the WHC was calculated using Equation 1:


(1) $$\begin{array}{l} WHC\left(g/g\right)=\frac{W_{1}-W_{0}}{W}, \end{array}$$

where *W*_1_ refers to the weight (g) of the EP tube before centrifugation; *W*_0_ represents the weight (g) of the EP tube without the supernatant; and *W* refers to sample weight.

#### 2.3.2. Oil-holding capacity

Using Zhang et al.'s method (22), the SDF (0.5 g) was put into olive oil (1:10, m/v) and kept at 23°C for 6 h. The mixture was centrifuged at 6,000 rpm for 20 min, and then, the collected sediment was weighed to calculate oil-holding capacity (OHC) as described in Equation 2:


(2) $$\begin{array}{l} OHC\left(g/g\right)=\frac{W_{1}-W_{0}}{W}, \end{array}$$

where *W*_1_ refers to the weight (g) of the EP tube before centrifugation; *W*_0_ represents the weight (*g*) of the EP tube without supernatant; and *W* refers to sample weight.

#### 2.3.3. Swelling capacity

Swelling capacity (SC) was measured based on Zhang et al.'s report (23). The SDF (0.2 g) and was mixed with distilled water (5 ml) to hydrate at 4°C for 18 h. The final SDF volume was observed to calculate SC using Equation 3:


(3) $$\begin{array}{l} SC\left(ml/g\right)=\frac{V}{W}, \end{array}$$

where *V* represents the final volume and *W* is the weight of SDF.

#### 2.3.4. Emulsifying activity and emulsion stability

First, the emulsion was prepared as follows: The SDF (2 g) was dispersed in deionized water and homogenized. Corn oil was added to the mixture and stirred for 2 min. The emulsion was then centrifuged at 1,500 rpm for 3 min (24). Emulsifying activity (EA) was calculated using Equation 4:


(4) $$\begin{array}{l} EA\left(ml/100ml\right)=\frac{V_{1}}{V}\times 100, \end{array}$$

where *V*_1_ and *V* are the volumes of the emulsified layer and total liquid, respectively.

After heating for 30 min at 80°C, the emulsion was cooled to 23°C. Emulsion stability (ES) was calculated using Equation 5:


(5) $$\begin{array}{l} ES\left(ml/100ml\right)=\frac{V_{1}}{V}\times 100, \end{array}$$

where *V*_1_ and *V* represent the emulsified layer volume and the total liquid volume, respectively.

#### 2.3.5. Least gelation concentration

Using Coffman et al.'s method (25), a series of SDF suspensions were prepared with the SDF from 2% to 12% (w/v), heated at 98°C for 60 min, and then kept in an ice bath for 60 min. When the suspensions were changed to a solid state even after inversion and shaking, it was recorded as the lowest concentration of the original SDF suspensions and referred to as the least gelation concentration (LGC).

#### 2.3.6. Rheological behavior

The soluble dietary fiber (1 g) was dissolved in deionized water (~25 ml), and the viscosity curve was tested using a Rheometer (Anton, MA, USA) at shear rates ranging from 0.1 to 1,000 s.

#### 2.3.7. Thermal analysis

Approximately 10 mg of SDF samples were mixed using thermogravimetric analysis/differential scanning calorimetry (TGA/DSC) and analyzed for thermal properties in the temperature range of 30–300°C. The heating rate was set at 5°C/min, and the flow rate of liquid nitrogen was 50 ml/min.

### 2.4. Functional properties

#### 2.4.1. Glucose adsorption capacity

Glucose adsorption capacity (GAC) was determined as described in a previous report (26). Briefly, the SDF sample (0.5 g) was mixed with 50–100 mmol/L glucose solution (50 ml) and incubated. After 6 h, the mixture was centrifuged, and then, the supernatant was collected to determine the reducing sugar content. GAC was calculated using Equation 6:


(6) $$\begin{array}{l} \text{GAC }\left(\text{mmol}/\text{g}\right)=\left[\left(A_{i}\;A\right)\times v\right]/m \end{array}$$

where *A*_*i*_ and *A* are the reduced sugar content without and with the SDF, g/100 g; *v* refers the solution volume, and *m* refers to SDF weight, g.

#### 2.4.2. Pancreatic lipase activity inhibition

Pancreatic lipase activity inhibition (PLAI) was tested using Chau's method (27). Briefly, 0.5 g of SDF, 1 ml of pancreatic lipase, 10 ml of soybean oil, and 50 ml of PBS were stirred for 60 min and then placed in an ice bath for 10 min. The mixture was titrated with NaOH (0.1 mol/L) using a phenolphthalein indicator (10 g/L). PLAI was calculated by using Equation 7:


(7) $$\begin{array}{l} \text{PLAI inhibition }(\%)\text{ = }[(V\;V1)\times C\times M]\text{/ }V\\ \;\times C\times M \end{array}$$

where *V* and *V*_1_ are the volumes of NaOH consumed without and with SDF, ml; *C* refers to NaOH concentration, mol/L; and *M* represents free fatty acid molar mass.

#### 2.4.3. Cholesterol adsorption capacity

Cholesterol adsorption capacity (CAC) was measured according to Jia et al. (13). Briefly, the yolks and deionized water were mixed at the ratio of 1:9 (v/v), and homogenized to obtain an emulsion, and the SDF sample (1 g) was added to 20 ml of the emulsion. Next, the emulsion was adjusted to pH 2.0 and 7.0, respectively, and incubated at 37°C for 0.5, 1, 1.5, or 3 h. Finally, the incubated emulsion was centrifuged at 6,200 rpm/min for 12 min. The CAC was calculated using Equation 8:


(8) $$\begin{array}{l} \text{CAC }\left(\text{mg}/\text{g}\right)=\left[\left(C_{y}\;C_{d}\right)\;\left(C_{y}\;C_{b}\right)\right]/W\times 20 \end{array}$$

where *W* is the SDF weight; *C*_*b*_ and *C*_*d*_ correspond to the cholesterol concentration in the emulsion.

#### 2.4.4. Nitrite ion adsorption capacity

Nitrite ion adsorption capacity was determined according to Zhu et al.'s method (28). At pH 2.0 and 7.0, 0.1 g of the SDF sample was mixed with 5 ml of NaNO_2_ solution (20 g/ml) and incubated at 37°C. After a 2-h incubation, the mixture was centrifuged, and the supernatant was moved into a tube and filled with 2.0 ml with deionized water. Finally, 2 ml of *p*-aminobenzene sulfonic acid solution (4 g/ml) and 1 ml/L naphthalene diamide hydrochloride solution (2 g/ml) were mixed to test NaNO_2_ concentration to calculate the value of NIAC using Equation 9:


(9) $$\begin{array}{l} \text{NIAC }\left(\text{μg}/\text{g}\right)\;=\;\left(m\;m_{1}\right)/w \end{array}$$

where *m* is NaNO_2_ weight before adsorption, *m*_1_ represents NaNO_2_ weight after adsorption, and *w* refers to the weight of the sample.

#### 2.4.5. Cation-exchange capacity

Using the method proposed by Huang and Ma (29), SDF (0.5 g) was dissolved in HCl, mixed, and incubated at 4°C for whole night. After filtration, the residue was thoroughly washed and titrated with AgNO_3_ solution. Finally, the residue was soaked and titrated with NaOH using phenolphthalein indicator. Cation-exchange capacity (CEC) was calculated using Equation (10):


(10) $$\begin{array}{l} \text{CEC }\left(\text{mmol}/\text{g}\right)=\left[\left(A_{i}\;A\right)\times v\right]/m \end{array}$$

where *A*_*i*_and *A* are the reduced H^+^ content without and with SDF; *v* refers to the consumed NaOH volume; and *m* represents SDF weight, g.

### 2.5. Structural characterization

#### 2.5.1. Scanning electron microscopy

Soluble dietary fiber samples were put on a specimen holder, sputter-coated with gold, and scanned with a scanning electron microscope at an accelerating voltage of 15.0 kV.

#### 2.5.2. Molecular weight determination

The molecular weight (Mw) of the SDF was determined using high-performance chromatography (30). The chromatographic column was a TSK-GEL column (8 mm × 300 mm), and the detector was a refractive index detector. The mobile phase was 0.05 M NaCl solution, and the eluted rate was 0.6 ml/min. The SDF sample was formulated in an aqueous solution (5 mg/ml) and filtered through a 0.22-μm filter. A standard dextran curve (dextrans of Mw of 1.27, 11.60, 80.90, 147.60, 273.00, 409.80, and 667.80 kDa) was prepared, and the molecular weight of SDFs was calculated.

#### 2.5.3. Monosaccharide composition

The monosaccharide composition of the SDF was determined using high-performance liquid chromatography (HPLC) (31). First, 0.01 g of the SDF was added to trifluoroacetic acid (TFA) (2 M, 2 ml) and hydrolyzed at 100°C for 8 h. After hydrolyzation, TFA was dried, washed with 1 ml of methanol, and then dissolved in 1 ml of distilled water. Next, the reaction solution was derivatized with 0.5 mol/L PMP-methanol solution and 0.3 mol/L NaOH for 1 h at 72°C. After cooling, the reacted product was neutralized with 0.3 mol/L HCl and chloroform. Finally, the mixture was centrifuged at a speed of 4,800 rpm for 10 min. Approximately 1 ml of chloroform was added in the process of absorbing the supernatant, and the procedure was run three times under the same conditions. The resulting supernatant was filtered through a biofilm and injected into the HPLC with 20 μl injection volume of a 0.1 mol/L of acetonitrile and PBS (pH = 6.7) mixture with a ratio of 18:82 at 1 ml/min flow rate. Standard solutions containing Rha, Man, GlcA, GalA, Glc, Gal, Xyl, and Ara were determined, as described earlier.

### 2.6. Statistical analysis

SPSS 20.0 was used as the software (SPSS, Inc., Chicago, IL, USA). All results were expressed as means ± standard deviation (SD). Data were subjected to analysis of variance (ANOVA), and significant differences (*p* < 0.05) of means were analyzed with Duncan's multiple range test.

## 3. Results

### 3.1. Proximate composition analysis

Table 1 presents the components of PS and SDFs. Compared with PS, the SDF prepared using the three methods was significantly decreased in impurities, including crude protein, crude fat, moisture, and ash, indicating that some treatments could significantly remove impurities, and microwave and PEF showed a more obvious effect of removing crude protein, with crude protein removal percentages of 68.3 and 78.4%, respectively. For other impurities, E-SDF and PEF showed the more obvious removing effects. Therefore, PEF-SDF had the minimum impurity content, which was in accordance with the purity of SDFs (the first row in Table 1). Of course, the yield was significantly increased in PEF-SDF. In addition, the total sugar content of prepared SDFs was higher than that of PS.

**Table 1:** Proximate composition of peanut shell (PS), enzymatic extraction (E-SDF), microwave extraction (M-SDF), and pulsed electric field extraction (PEF-SDF)^1^.

**Proximate composition (g/100 g)**	**PS**	**SDFs**		
		**E-SDF**	**M-SDF**	**PEF-SDF**
Dietary fiber	83.91 ± 0.65^2a^	95.27 ± 0.57^3c^	93.54 ± 0.75^b^	96.02 ± 0.48^c^
Crude protein	5.89 ± 0.17^a^	2.19 ± 0.24^b^	1.87 ± 0.11^c^	1.27 ± 0.14^d^
Crude fat	2.45 ± 0.32^a^	1.08 ± 0.04^c^	1.45 ± 0.07^b^	0.93 ± 0.07^c^
Moisture	2.84 ± 0.11^a^	1.04 ± 0.07^c^	1.41 ± 0.12^b^	1.01 ± 0.05^c^
Ash	1.78 ± 0.04^a^	0.98 ± 0.02^c^	1.24 ± 0.03^b^	0.95 ± 0.02^c^
Total sugar	11.25 ± 0.25^a^	27.36 ± 0.19^b^	35.87 ± 0.64^d^	33.18 ± 0.27^c^
Yield^4^ (%)	–	20.14 ± 0.36^a^	22.75 ± 0.12^b^	23.82 ± 0.45^b^

^1^The values represent means of triplicates ± standard deviation (SD). Values in the same row with different letters are significantly different (p < 0.05).

^2^Refers to DF extraction yield from PS.

^3^Refers to soluble dietary fiber (SDF) content of extracts.

^4^Refers to SDF extraction yield from PS.

### 3.2. Physicochemical properties

The physicochemical properties were better represented in SDFs than in PS, suggesting that PS contains large amounts of IDF.

#### 3.2.1. WHC, SC, and OHC

As described in Table 2, the WHC of PEF-SDF (5.67 ± 0.67 g/g) was significantly higher than that of M-SDF (4.35 ± 0.19 g/g) and E-SDF (3.98 ± 0.29 g/g; *p* < 0.05). In contrast to WHC, PEF showed no significant advantage in SE and OHC compared to microwaves. The SC of PEF-SDF (6.96 ± 0.88 ml/g) and M-SDF (6.75 ± 0.73 ml/g) was significantly increased (*p* < 0.05) compared to that of E-SDF (4.57 ± 0.36 ml/g) and PS (3.09 ± 0.29 ml/g; *p* < 0.05). The OHC of PEF-SDF (3.89 ± 0.41 g/g) and M-SDF (3.57 ± 0.18 g/g) was significantly increased (*p* < 0.05) compared with that of E-SDF (2.88 ± 0.20 g/g) and PS (1.88 ± 0.19 ml/g). In contrast, a decreasing trend in LGC can be observed from 11.26 ± 0.71% (E-SDF) to 8.18 ± 0.28% (PEF-SDF; *p* < 0.05). The results showed that PEF-SDF has better gelation properties (32).

**Table 2:** The physicochemical properties^1^ of PS, E-SDF, M-SDF, and PEF-SDF.

**Samples**	**WHC** **(g/g)**	**OHC** **(g/g)**	**SC** **(ml/g)**	**LGC** **(%)**	**EA** **(ml/100 ml)**	**ES** **(ml/100 ml)**
PS	1.26 ± 0.31^a^	1.88 ± 0.19^a^	3.09 ± 0.29^a^	ND	ND	ND
E-SDF	3.98 ± 0.29^b^	2.88 ± 0.20^b^	4.57 ± 0.36^b^	11.26 ± 0.71^a^	66.37 ± 1.83^a^	55.11 ± 1.13^a^
M-SDF	4.35 ± 0.19^c^	3.57 ± 0.18^c^	6.75 ± 0.73^c^	9.69 ± 0.94^b^	72.35 ± 1.69^b^	65.23 ± 1.75^b^
PEF-SDF	5.67 ± 0.67^d^	3.89 ± 0.41^c^	6.96 ± 0.88^c^	8.18 ± 0.28^c^	79.69 ± 2.36^c^	70.36 ± 2.13^c^

^1^The values represent means of triplicates ± SD. Values in the same column with different letters are significantly different (p < 0.05).

ND, not determined; WHC, water-holding capacity; OHC, oil-holding capacity; SC, swelling capacity; EA, emulsifying activity; ES, emulsion stability; LGC, least gelation concentration.

#### 3.2.2. EA, ES, and LGC

Based on the results in Table 2, the EA values were 66.37 ± 1.83, 72.35 ± 1.69, and 79.69 ± 2.36 ml/100 ml for E-SDF, M-SDF, and PEF-SDF, respectively, with a clear increasing trend from E-SDF to PEF-SDF (*p* < 0.05). Similarly, PEF-SDF showed the largest ES value, which indicates that PEF-SDF can be regarded as a better emulsifier.

#### 3.2.3. Rheological behavior

**Figure 2:**
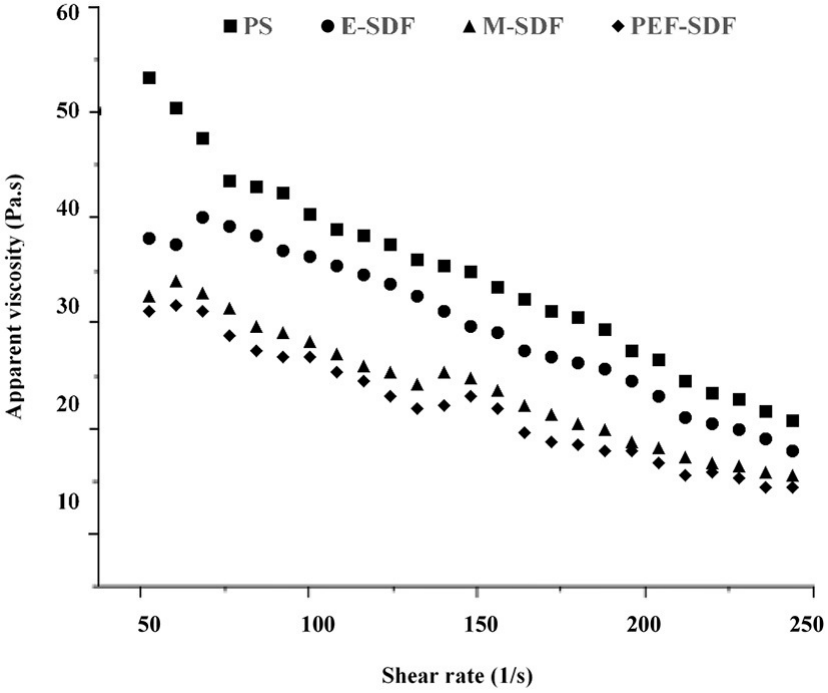
The dependence of apparent viscosity on the shear rate for of aqueous solution of peanut shell (PS), enzymatic extraction (E-SDF), microwave extraction (M-SDF), and pulsed electric field extraction (PEF-SDF) at concentration of 100 mg/L at 25°C.

The rheological behavior is shown in Figure 2. With a rise in the shear rate, a decrease in viscosity is shown, indicating a sign of shear-thinning behavior, which was speculated to be a pseudoplastic fluid (33). In addition, the initial apparent viscosity of SDFs at a concentration of 100 mg/L was observed to decrease slightly from the raw material PS with an increasing shear rate. It was reported that the apparent viscosity was associated with the molecular chain arrangement, which could be speculated that SDFs decreased the entanglement with increasing shear rate. Moreover, PS appeared to be a tight network that tended to be more stable than SDFs at a high shear rate (34, 35). However, a sharp reduction in the apparent viscosity of PS is shown as the shear rate increased, as a result of disruption of the entanglement by the application of shear to align the molecules in the direction of flow (36).

#### 3.2.4. Thermal analysis

**Figure 3:**
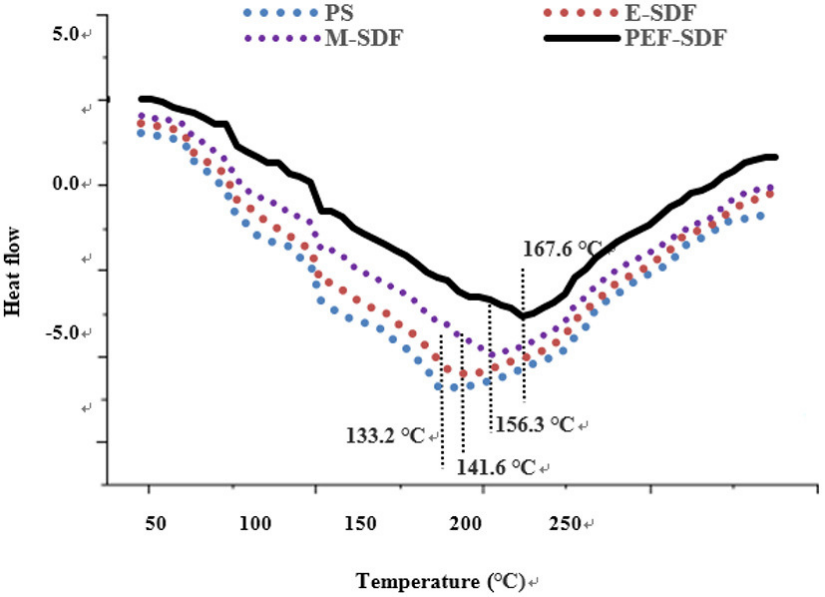
The thermodynamic characteristics of SDF obtained from different extraction methods.

Figure 3 shows that the peak temperature during E-SDF, M-SDF, and PEF-SDF (141.6, 156.3, and 167.6°C) was higher than that of PS (133.2°C). The difference in peak temperature indicates that more energy is needed to decompose SDF; this finding was in accordance with the SDF from soybean residues (37). The higher peak temperatures of M-SDF and PEF-SDF were due to the abundance of short chains, which could explain that SDF had strong hydrogen bonds that required substantial energy to destroy its crystalline structure (38). In addition, when heated to 240°C, heat absorption and heat release are balanced, suggesting high thermal stability.

### 3.3. Functional properties

#### 3.3.1. GAC, CAC, and NIAC

As shown in Table 3, SDFs from different extraction methods have a higher GAC value than PS (*p* < 0.05). Furthermore, PEF-SDF exhibited the highest GAC value, suggesting higher glucose levels (39), resulting in a reduced glycemic reaction. Taken together, the CAC values of E-SDF, M-SDF, and PEF-SDF were significantly increased compared with PS (*p* < 0.05), consistent with a previous report (40). In different SDFs, the CAC value of SDF belonging to the PEF-SDF was the largest (52.63 ± 2.01%) and that belonging to E-SDF was the smallest (32.94 ± 1.92%) in a simulated small-intestinal environment (pH = 7.0). In addition, the NIAC capacity of PS exhibited lower NO${}_{2}^{-}$ scavenging ability than the three types of SDFs (*p* < 0.05). A similar trend was observed for GAC and CAC, with NIAC increasing significantly from E-SDF to PEF-SDF (*p* < 0.05).

**Table 3:** The functional properties^1^ of PS, E-SDF, M-SDF, and PEF-SDF.

**Samples**	**Glucose adsorption capacity** **(GAC, mmol/g)**	**Pancreatic lipase inhibition capacity** **(PLIC, %)**	**Cholesterol adsorption capacity** **(CAC, %)**	**Cation-exchange capacity** **(CEC, mmol/g)**	**Nitrite ion adsorption capacity** **(NIAC, μg/g)**
PS	2.58 ± 0.34^a^	0.73 ± 0.02^a^	25.84 ± 1.56^a^	0.63 ± 0.03^a^	148.69 ± 3.65^a^
E-SDF	3.19 ± 0.25^b^	0.71 ± 0.03^a^	32.94 ± 1.92^b^	0.94 ± 0.02^b^	186.35 ± 4.01^b^
M-SDF	3.95 ± 0.41^c^	0.86 ± 0.06^b^	46.28 ± 1.85^c^	1.58 ± 0.08^c^	214.28 ± 3.95^c^
PEF-SDF	4.67 ± 0.39^d^	0.98 ± 0.05^c^	52.63 ± 2.01^d^	1.76 ± 0.14^d^	246.10 ± 4.19^d^

^1^The values represent means of triplicates ± SD. Values in the same column with different letters are significantly different (p < 0.05).

#### 3.3.2. PLAI and CEC

Table 3 shows the addition of PS or SDFs to cause an inhibitory effect on lipase activity. PS and E-SDF showed no significant difference in the inhibit pancreatic capacity (*p* > 0.05). In contrast, there were clear differences among E-SDF, M-SDF, and PEF-SDF (*p* < 0.05), with PEF-SDF having the strongest inhibitory effect.

Compared with PS, the CEC values of SDFs were clearly increased from 0.94 ± 0.02 (E-SDF) to 1.76 ± 0.14 mmol/g (PEF-SDF; *p* < 0.05; Table 3), which were 1.49 (E-SDF), 2.51 (W-SDF), and 2.79 (PEF-SDF) times larger than PS.

### 3.4. Structural analysis

#### 3.4.1. Scanning electron morphology

**Figure 4:**
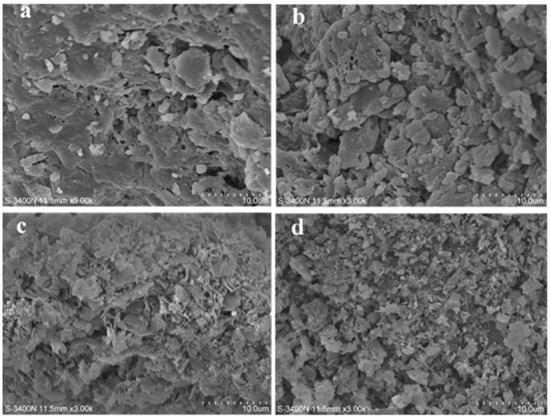
The scanning electron microcopy (SEM) images of PS **(a)**, E-SDF **(b)**, M-SDF **(c)**, and PEF-SDF **(d)**.

Figures 4a–d show the network structure of PS and SDFs, with significance in the morphologies of PS, E-SDF, M-SDF, and PEF-SDF. PS was compact and unevenly packed with particles of different sizes, and E-SDF exhibited a compact texture coated with a wrinkle along with cracks and holes. However, M-SDF and PEF-SDF had looser and more porous surfaces.

#### 3.4.2. Monosaccharide composition

Monosaccharides are assessed and are listed in Table 4. The total sugar content of the PS and SDF samples was more than 95% (w/w), while the SDF samples (65.15% for E-SDF, 74.08% for M-SDF, and 77.26% for PEF-SDF) were dominated by neutral sugars compared with the PS (60.63%). Table 4 summarizes PS and SDF samples, which were mainly composed of rhamnose, glucose, arabinose, xylose, galactose, mannose, and galacturonic acid. The major monosaccharides were glacturonic acid, xylose, and galactose. Although the monosaccharide composition of the SDFs was similar to that of the PS raw material, the contents of arabinose, galactose, glucose, mannose, and xylose increased significantly.

**Table 4:** The monosaccharide composition of PS, E-SDF, W-SDF, and PEF-SDF.

**Monosaccharide**	**PS**	**E-SDF**	**M-SDF**	**PEF-SDF**
Rhamnose (Rha)^1^	0.855 ± 0.050^2^	0.791 ± 0.062	0.807 ± 0.051	0.848 ± 0.063
Arabinose (Ara)	4.221 ± 0.271	4.562 ± 0.241	4.742 ± 0.202	5.222 ± 0.351
Galactose (Gal)	7.892 ± 0.871	10.527 ± 1.432	10.82 ± 1.312	11.272 ± 0.972
Glucose (Glu)	6.029 ± 0.564	8.441 ± 0.784	9.962 ± 0.972	10.082 ± 0.941
Xylose (Xyl)	8.923 ± 0.882	10.43 ± 1.111	12.152 ± 1.122	10.962 ± 1.105
Mannose (Man)	4.481 ± 0.442	5.961 ± 0.892	6.771 ± 0.918	6.361 ± 0.832
Fructose (Fru)	3.352 ± 0.441	3.432 ± 0.662	3.731 ± 0.462	3.512 ± 0.431
Galacturonic acid (GalA)	17.821 ± 1.112	16.912 ± 0.982	11.372 ± 0.963	10.432 ± 0.832
R1	1.329 ± 0.088	1.068 ± 0.040	0.708 ± 0.034	0.555 ± 0.054
R2	0.054 ± 0.017	0.047 ± 0.012	0.074 ± 0.027	0.084 ± 0.022
R3	25.84 ± 1.211	19.519 ± 1.385	19.442 ± 2.983	19.64 ± 2.010
HG = GalA – Rha	16.722 ± 1.039	16.21 ± 1.112	10.265 ± 1.103	9.584 ± 1.043
RG-I = 2 Rha + Ara + Gal	13.334 ± 1.412	16.688 ± 2.515	17.545 ± 2.415	18.126 ± 2.510
HG/RG-I	1.27 ± 0.089	0.96 ± 0.109	0.69 ± 0.121	0.541 ± 0.086

^1^mol %.

^2^The values represent means of triplicates ± SD.

R1 = GalA/(Rha + Ara + Gal); R2 = Rha/GalA; R3 = (Ara + Gal)/Rha.

The molecular structure was modeled by using sugar molar ratios. The relatively higher homogalacturonan (HG) in PS suggested that the PS was predominantly composed of HG as a main building block. Compared with PS and E-SDF, M-SDF and PEF-SDF contained more neutral sugars and small proportions of HG regions, indicating less linearity and more branching, suggesting that these were similar to a previous report (41). Correspondingly, a clear decrease in R1 and HG/RG-I and a significant increase in R2 highlight the prevalence of linear segments in the structure of PS and E-SDF, whereas M-SDF and PEF-SDF exhibited higher levels of branching (42). HG/RG-I (the ratio of homogalacturonan/rhamnogalacturonan) was calculated as 1.27, which shows that PS has a homogalacturonan-rich (linear) structure, while the SDF sample showed a rhamnogalacturonan structure. The lower ratio of Rha to GalA (R2) in PS and E-SDF indicated the contribution of RG-I blocks within the polysaccharide backbone, indicating that the samples contained only a small proportion of RG-I segments (43). The ratio of RG-I segments was relatively high (Ara + Gal)/Rha (R3) in PS samples, approximately indicating the larger degree of RG-1 segments with a longer average length of side chains than in SDF samples (44).

#### 3.4.3. Molecular weight

Soluble dietary fibers prepared by different extraction methods had different molecular weights. As shown in Table 5, compared with PS, enzymatic, microwave, and PEF treatments significantly decreased the molecular size. PEF-SDF had the smallest average molecular weight, and in addition, the results indicate that SDFs based on PEF treatment exhibited a narrower polydispersity.

**Table 5:** The effects of PS, E-SDF, W-SDF, and PEF-SDF on molecular weight^1^.

**Sample**	**Weight-average molecular weight** **Mw (kDa)**	**Number-average molecular weight** **Mn (kDa)**	**Polydispersity** **Pd (Mw/Mn)**
PS	486	173	2.81
E-SDF	201	112	1.79
M-SDF	152	101	1.50
PEF-SDF	136	96	1.42

^1^Values are given as means of independent experiments.

## 4. Discussion

The DF yield of material PS was 83.91% (Table 1) and higher than that of pear (57 g/100 g) (45) and rice bran (27 g/100 g) (15), which indicates that PS is a promising source of DF. Therefore, full utilization of PS can improve the preparation of DF used in functional food production and reduce waste and contamination of peanut by-products. Therefore, it is a sustainable industry.

Compared with PS, SDFs showed a significant improvement in physicochemical and functional properties, partly due to structural modifications (46) and partly due to composition. PS included SDF and IDF, and IDF was the major. Because of the solubility of IDF, its physicochemical and functional properties were relatively poor. In the intramolecular structure, the larger the molecular weight of PS, the greater the degree of linearity and the length of the side chains (Table 4). In network structures, PS showed heterogeneity, appearing as compact and unevenly packed particles of different sizes (Figure 4), which was in accordance with it having the largest polydispersity (Table 5). The increase in total sugar production indicated that the treatment induced the conversion of insoluble fibers to SDF, such as the degradation of cellulose and hemicellulose, which was consistent with previous studies (47, 48).

M-SDF and PEF-SDF showed relatively clear improvement effects in the physicochemical properties including WHC, OHC, SC, LGC, EA, and ES, the unifying phenomenon of the most effective treatment belonging to PEF followed by microwave and enzymatic can be observed, with M-SDF and PEF-SDF, showing significance in WHC, LGC, EA, and ES. Water-related properties, including WHC and SC, have been reported to be associated with DF individual components, including molecular size and structure (network density and porosity) (46). It was speculated that the larger the WHC and SC values of PEF-SDF and M-SDF, the greater the proportion of short-chain dietary fiber (32, 49). Similarly, the higher OHC values of PEF-SDF and M-SDF might be associated with more complex porous structures and surface areas, which is consistent with a previous report (50). This structural change was reflected in the monosaccharide composition. After pretreatment, the contents of arabinose, galactose, glucose, mannose, and xylose increased significantly, which indicated that certain pretreatments could degrade most of the cellulose to release some of the hemicelluloses and then partly decompose the hemicellulose (48). Especially, a previous report speculated that microwave and PEF treatments might transform the structure of the molecules (21). Why did microwave and PEF treatments change the molecular structure? It was speculated that microwaves might disrupt the cross-links between polysaccharide molecules (51) and may have enhanced physicochemical properties (52). The high voltage of PEF could destroy cellulose and hemicellulose molecular chains, leading to a reduction in molecular polymerization (53). Meanwhile, PEF was approved to induce a high porous structure (54), and the higher porosity of PEF-SDF (reflected in Figure 3) led to more exposure to hydrogen bonds and water-binding sites. Such special structural changes might be due to the increase in certain bonds and intermolecular forces by PEF, including hydrogen bonds and hydrophobic interactions that could improve viscosity and viscoelasticity (43, 55) and even physicochemical functional properties.

Next, some chemical reaction tests showed that SDFs had better functional properties than PS. The direct effect of PS-rich IDF is mainly to promote the growth of probiotics. Of course, the stronger capacity of GAC, CAC and NIAC of PEF-SDF is due to its structure. PEF-SDF showed a higher degree of porous network connection and larger surface area, resulting in larger adsorption of glucose in part (56), which leads to a lower more potent glycemic reaction (12, 57), and this effect was reported to be negatively associated with diabetes risk (57). PEF-SDF had a higher WHC appearance in the amorphous state, and fewer microcrystalline bundles appear in the amorphous state, leading to more exposed active groups that can directly chelate cholesterol molecules. Meanwhile, a higher SC of PEF-SDF was more likely to gelatinize (Table 2) and bind to cholesterol, resulting in reduced absorption (58). In addition, PEF-SDF showed the strongest PLIC, partly because of its porous structure. According to reports, PEF can increase the specific surface area, effectively embedding the oil while inhibiting pancreatic lipase capacity (59).

Based on the abovementioned analysis, good physicochemical properties can affirm better application processing. Based on the enhanced physicochemical properties of PEF-SDF and M-SDF, they will possess an increase in the content of SDFs for potential health and the power of convenient applications.

M-SDF showed significant improvements in physicochemical and functional properties compared with PS, although the effects remained to be slighter when compared with M-SDF and PEF-SDF. E-SDF also exhibited special advantages. The E-SDF showed brighter colors and higher purity than those of the SDF prepared by the SDF's physical preparation methods (M-SDF and PEF-SDF). For practical production, the clear disadvantages of biological methods are high cost, difficulty in optimizing fermentation conditions, long operating cycles, and susceptibility to contamination by other microorganisms, while microwave and PEF showed high efficiency, low costs, and easier operation. In nonthermal technology, PEF showed a mild modification for better physicochemical and functional properties due to the structural changes in SDF. SDF treated with PEF with a looser spatial structure had a higher specific surface area, which might improve the ability to adsorb or bind several molecules, including water, oil, and nitrite ions (60, 61), which were approved for pulsed electric field-modified dietary fibers from orange peel (32). Another mild enzymatic modification shows unique advantages such as specificity and localization. To make the best use of modified SDFs in functional foods, an integrated modification process with multiple methods, such as a combination of enzymatic and PEF methods, will be explored in the future.

## 5. Conclusion

In this study, enzyme-, microwave-, and pulsed electric field extraction methods were employed to prepare SDFs from PS, and their structural, flow behavior, physicochemical, and functional properties were investigated. The results showed that the pretreatment could improve the physicochemical and functional properties of PS. PEF-SDF and M-SDF possessed a more complex structure and higher thermal stability than E-SDF. Notably, PEF-SDF showed the lowest molecular weight, strongest gelation properties, OHC, WHC, SC, EA, ES, CAC, GAC, CEC, and PLAL. This finding will affirm a promising technology for the preparation of SDF from peanut by-products into good raw materials for functional foods. The eco-friendly utilization use behavior of PS can significantly decrease by-product waste and indirectly result in substantial income growth, which will ultimately improve people's health. Therefore, this behavior will lead to sustainable development for the world.

## Data availability statement

The raw data supporting the conclusions of this article will be made available by the authors, without undue reservation.

## Author contributions

Conceptualization: RF, LW, and BZ. Methodology: RF, XW, and LW. Investigation: LW, YY, and SY. Writing—original draft preparation: LW and RF. Writing—review and editing: RF and BZ. All authors contributed to the article and approved the submitted version.
